# Barriers to uptake of cervical cancer screening among women in Nigeria: a systematic review

**DOI:** 10.4314/ahs.v22i2.33

**Published:** 2022-06

**Authors:** Joy J Mafiana, Sushma Dhital, Mohamednour Halabia, Xiaohui Wang

**Affiliations:** School of Public Health, Lanzhou University, Lanzhou 730000, China

**Keywords:** Cervical cancer screening, barriers, uptake, Nigeria, systematic review

## Abstract

**Background:**

Cervical cancer is the second most frequent cancer and cause of cancer-related deaths among women in Nigeria. The Visual inspection with acetic acid and cryotherapy “see and treat” screening approach is a feasible and effective method that can be implemented in low resource settings like Nigeria; however, screening utilization is still low.

**Objective:**

This systematic review aims at offering a comprehensive synthesis of studies that assessed the barriers preventing women from utilizing cervical cancer screening services in Nigeria.

**Methods:**

Electronic data search was performed on PubMed, Cochrane Library, EMbase, Directory of Open Access Journals, Google Scholar, and ScienceDirect, and quality assessment was conducted for the included studies. Data were extracted independently by two authors and thematically analysed for barriers to cervical cancer screening utilization.

**Results:**

Fifteen studies, consisting of 9,995 women aged 15 and above published between 2007 and 2020, were included. Frequently reported barriers to cervical screening include lack of knowledge of cervical cancer and screening, health service factors, screening is unnecessary, fear of outcome and procedure, and financial constraints.

**Conclusion:**

Lack of adequate information about cervical cancer is a significant hindrance to screening; this factor is strongly associated with the numerous misconceptions and negative perceptions. The study highlights the need for further assessment of the sociodemographic determinants of cervical cancer screening uptake in Nigeria. Preventive strategies should be targeted at improving the dissemination of valid information, reducing the knowledge gap among women, and addressing the financial and health service factors.

## Introduction

Cervical cancer constitutes a significant public health problem and ranks the fourth most common cause of cancer incidence and mortality in women worldwide^1^. Although a decline has been observed in its ranking globally, there is still an upsurge in the incidence and mortality rate. It was the second most common cancer in 2000 with 468,000 new cases and 233,000 deaths^2^; in 2008, it ranked third with 530,000 cases and 275,000 deaths^3^; while in 2018, over 570,000 cases and 311,000 deaths occurred globally^4^. Based on current statistics, most regions of the world have experienced a decline in the incidence of cervical cancer^5^. Conversely, it is still a leading cause of cancer-related death among women in Western Africa, with an approximate estimate of 84% cases and 88% deaths^4^.

In Nigeria, it is the second most frequent cancer and cause of cancer-related deaths among women^6^. Current estimates indicate that every year 14,943 women are diagnosed with cervical cancer, and 10,403 die from the disease in Nigeria^7^. In 2018, it accounted for 12.9% incidence and 14.8% mortality of cancer cases^8^. In furtherance, it was reported that there were 12,075 new cases and 7,968 deaths in 2020^9^. In terms of risks of exposure, it is estimated that over 50.33 million women aged 15 years and above are at risk of developing the disease in Nigeria^7^.

Cervical cancer develops when abnormal cells in the lining of the cervix grow uncontrollably^10^. Over 99% of cervical cancer cases have been attributed to infection with sexually transmitted high-risk Human papillomaviruses (HPV)1. Globally, HPV 16 and 18 have been linked to over 70% of all cervical cancer cases^6^. It is estimated that about 3.5% of women in Nigeria harbour HPV-16/18 infection at a given time^7^. According to the World Health Organization (2020a), cervical cancer can be eliminated within a generation via a comprehensive approach consisting of three interdependent evidence-based interventions to reduce the burden of the disease. Screening for and treating pre-cancer is a secondary approach targeted at asymptomatic women aged 30–49 years (or ages determined by national standards) to identify precancerous lesions. The goal is to decrease the incidence and mortality associated with cervical cancer by intercepting the progress from pre-cancer to invasive cancer. The recommended screening methods are; HPV testing, Papanicolaou (Pap) smear or Cytology and Visual inspection with acetic acid (VIA)^11^.

Based on the findings of the World Health Organization (2012) study, VIA and cryotherapy “see and treat” or “single visit” approach is a feasible and effective method that can be implemented in countries with low resource settings like Nigeria. It ensures adherence to treatment soon after diagnosis and can be implemented in a primary healthcare facility^13^. This approach has been implemented in some North African countries like Morocco^14^ which had 3,388 new cases and 2,465 deaths in 2019 15 compared to the 12,075 new cases and 7,968 deaths in Nigeria^9^. However, Nigeria is still faced with the challenge of low uptake of cervical screening and treatment of precancerous lesions. Hence, women at the precancer stage are undiagnosed but later detected at advanced stages of invasive cervical cancer. A similar case was observed in Oguntayo et al.^16^ study, where 78% of the patients diagnosed with cervical cancer were at the third stage of the disease. Sequel to this background, it is imperative to understand the factors inhibiting women from utilizing cervical screening services. To our knowledge, no previous systematic review on barriers to the uptake of cervical cancer screening among women in Nigeria has been conducted. Therefore, the review aims at offering a comprehensive synthesis of studies that assessed the barriers preventing women from utilizing cervical cancer screening services in Nigeria. Additionally, provide an overview and better understanding of the issues across the country's six geopolitical zones.

## Methods

### Eligibility criteria

We followed the Preferred Reporting Items for Systematic Reviews and Meta-analyses (PRISMA) guidelines^17^ to perform a systematic review of the barriers to uptake of cervical cancer screening among women in Nigeria.

### Inclusion criteria

Primary quantitative, qualitative, and mixed method studies that were published in English and examined the uptake and barriers to cervical cancer screening among women in Nigeria were included. Qualitative studies were included to generate information free from researchers' preconceived expectations, while quantitative studies were included to identify associations between various factors and screening uptake.

### Exclusion criteria

Studies excluded in this review were prevalence studies; study protocols; policy documents; cross-sectional studies examining only knowledge score, perceived susceptibility, and risk; studies that assessed barriers to HPV vaccination; and those that did not address barriers to cervical cancer screening uptake. Studies that focused on barriers faced by women with HIV were not included because the challenges encountered by this group of women are unique. Studies that described the views of healthcare workers were excluded to avoid bias in reporting healthcare-related barriers, and studies that described the views of men were also excluded. Studies published prior to 2007 were excluded to capture current research reports sequel to the establishment of the Nigeria Health Insurance Scheme (NHIS) in 2005 and the National Health Policy Reform programme of 2004–2007. In addition, studies not published in English or not available in full text, were also excluded.

### Search methods for identification of studies

The literature search for this study was performed using PubMed, The Cochrane Library, EMbase, Directory of Open Access Journals (DOAJ), Google Scholar, and ScienceDirect in April 2020. The search terms included ‘cervical cancer’, ‘cancer of the cervix’, ‘cervical neoplasms’, ‘cervical cancer screening’, ‘HPV testing’, ‘pap smear’, ‘visual inspection with acetic acid’, ‘barriers’, ‘factors’, ‘limitations’, ‘uptake’, ‘utilization’, ‘Nigeria’. The search terms were performed separately in all databases and then combined with ‘OR’ and ‘AND’ operators. For example (‘cervical cancer’ OR ‘cancer of the cervix’ OR ‘cervical neoplasms’) AND (‘cervical cancer screening’ OR ‘HPV testing’ OR ‘Pap Smear’ OR ‘visual inspection with acetic acid’) AND (‘barriers’ OR ‘limitations’ OR ‘factors’) AND (‘uptake’ OR ‘utilization’) AND (Nigeria). The database search was supplemented by manually examining reference lists of included articles and was completed in September 2020. The search was limited to the year 2007 onwards.

### Study selection

Upon data search, three authors independently screened the titles and abstracts of the articles, following these; the full text of the articles identified was independently reviewed by two authors, and discrepancies were resolved by consensus.

### Data extraction

Data were extracted independently from the included studies by two of the authors using a jointly developed, piloted, and revised data-extraction form, and discrepancies were resolved by consensus. The basic information of the studies (author; title; date of publication, study aim; location: region/state; study setting, research methods; study participants; and sample size), the proportion of women that had undergone cervical cancer screening, and the barriers to cervical cancer screening utilization were extracted. The proportion of women under each category of the identified barriers were also extracted for quantitative studies.

### Quality assessment of included studies

For quality assessment, the study used the appraisal method designed by Sirriyeh et al. (2012) for studies with diverse designs. The tool consists of 16 criteria with a fourpoint scale used in assessing the overall quality of mixed qualitative and quantitative data. Codes were allocated for the components in the checklist; 0 = Not at all; 1 = Very slightly; 2 = Moderately; and 3 = Complete. Given the small number of the included studies, a minimum of 2 score points is imperative for the second criteria of the assessment checklist.

### Data analysis

Due to the heterogeneity of the included studies, data from the studies were thematically analysed for barriers to cervical cancer screening utilization.

## Results

### Study search outcome

**Figure 1:**
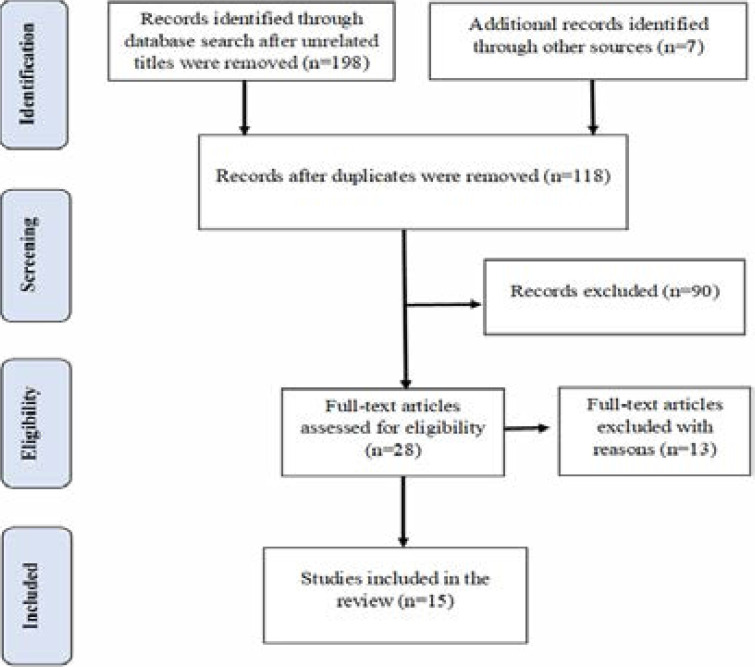
Flow diagram of the study selection process adapted from PRISMA Guidelines.

Figure 1 shows the selection process of the articles retrieved. The initial database search returned 198 articles after unrelated titles were removed, and 7 additional articles were obtained from reference lists. Eighty-seven duplicate articles were removed, and the abstracts of 118 were read, and 90 were excluded for not meeting the inclusion criteria. Following full-text review, 13 additional articles were excluded, as they did not specifically address barriers to cervical cancer screening reported by women only.

### Study characteristics

**Figure 2:**
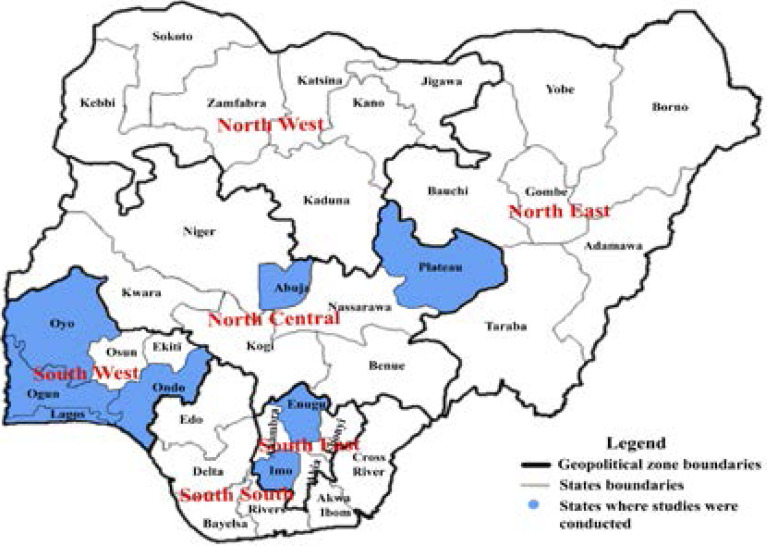
Map of Nigeria showing the states and geopolitical zones where the included studies were conducted

Table 1 provides information on the author, publication year, geographical zone/study site, study setting, study participants, sample size, research methods, and the proportion of women screened in the studies (if measured). Fifteen studies were included in the final analysis, 12 were cross-sectional studies, 2 were qualitative studies using focus group discussions (FGDs), and 1 was a mixed-method study. Four of the studies were community-based, 8 were health facility-based, 1 was conducted in tertiary institutions (education), 1 in religious instiutions (Christian), and 1 in federal non-healthcare establishments. Eight studies were conducted in the Southwestern geopolitical zones, 4 in the Southeast, 2 in the North-central, while one was conducted in both the Southwest and North-central region (Figure 2). The studies included 9,995 women aged 15 and above; 5,044 from health facilities, 3,350 were recruited from households within the study location, 815 from religious institutions, 398 from tertiary institutions, and 388 from federal non-healthcare establishments. The proportion of women that had utilized cervical cancer screening services were measured in 14 studies and ranged from 1.4% to 38.8%. The studies were published between 2007 and 2020.

**Table 1:** Characteristics of Included Studies

Author/year	Region/study site	Study setting	Sample size	Study design and instrument	Screened proportion
Ndikom and Ofi (2012)	Southwest/Ibadan, Oyo State	Health facility	82	Qualitative/FGD	NS
Titiloye et al. (2017)	Southwest/Ondo town, Ondo State	Community based	244	Cross-sectional/Questionnaire	28(15.6%)
Abiodun et al. (2013)	Southwest/across 20 LGA, Ogun State	Community based	2000	Cross-sectional/Questionnaire	27(1.4%)
Isa Modibbo et al. (2016)	North-central/Abuja and Southwest/Ondo State	Health facility	49	Qualitive/FGD	19(38.8)
Amu et al. (2017)	Southwest/Somolu LGA, Lagos State	Community based	260	Cross-sectional/Questionnaire	26 (10%)
Chigbu and Aniebue. (2011)	Southeast/Enugu State	Health facility	3712	Cross-sectional/Questionnaire	389(10.5%)
Nwankwo et al. (2011)	Southeast/Ugbawka, Ozalla and Ijinike communities, Enugu State	Religious institution	815	Cross-sectional/Questionnaire	34(4.2%)
Ogwunga et al. (2020)	Southeast/Imo State	Educational institution	398	Mixed method/Questionnaire	17(4.3%)
Ezem (2007)	Southeast/Owerri, Imo State	Community based	846	Cross-sectional/Questionnaire	60(7.1%)
Amos and Awolude (2019)	Southwest/Ibadan, Oyo State	Health facility	85	Cross-sectional/Questionnaire	23(27.1%)
Bammeke and Ndikom (2014)	Southwest/Ibadan, Oyo State	Health facility	100	Cross-sectional/Questionnaire	5%
Hyacinth et al. (2012)	North-central/Jos, Plateau State	Federal non-healthcare establishment	388	Cross-sectional/Questionnaire	34(10.2%)
Leo et al. (2020)	North-central/Abuja	Health facility	289	Cross-sectional/Questionnaire	105(36.3%)
Okunowo and Smith- Okonu (2020)	Southwest/Surulere LGA, Lagos State	Health facility	522	Cross-sectional/Questionnaire	92(18.4%)
Ndikom, Ajibade, and Oluwasola (2020)	Southwest/Ibadan, Oyo State	Health facility	205	Cross-sectional/Questionnaire	33(16.1%)

NS = Not specified

### Quality assessment of studies

Table 2 summarizes the quality assessment of the studies. The assessment scores ranged from 16–27 for quantitative studies, 18–30 for qualitative studies, and 18 for the mixed-method study. Only 2 of the included studies were based on a theoretical framework. There was a clear description of the research setting and objectives in all the studies. The evidence of sample size considered was explained in 10 studies. Procedures for data collection were described in all the studies, while 13 stated the rationale for data collection tools.

**Table 2:** Quality assessment of included studies using the Sirriyeh et al. (2012) tool for diverse study designs

Author/year	1	2	3	4	5	6	7	8	9	10	11	12	13	14	15	16	Total Score
Ndikom and Ofi (2012)	3	3	3	0	1	3	1	2	N/A	N/A	0	0	1	0	0	1	18
Titiloye et al. (2017)	0	3	3	2	2	2	0	2	0	0	N/A	1	1	N/A	0	0	16
Abiodun et al. (2013)	0	2	2	2	3	2	3	3	1	0	N/A	0	0	N/A	2	0	20
Isa Modibbo et al. (2016)	0	3	3	2	1	3	2	3	N/A	N/A	0	3	3	3	2	2	30
Amu et al (2017)	0	3	3	2	3	2	1	3	2	0	N/A	1	2	N/A	2	0	24
Chigbu and Aniebue (2011)	0	3	3	0	2	1	1	2	2	0	N/A	3	2	N/A	2	0	21
Nwankwo et al. (2011)	0	3	3	0	2	3	3	3	3	0	N/A	0	2	N/A	3	2	27
Ogwunga et al. (2020)	0	3	3	0	1	3	1	3	0	0	0	0	2	2	0	0	18
Ezem (2007)	0	3	2	1	2	2	0	2	2	0	N/A	0	0	N/A	2	0	16
Amos and Awolude (2019)	0	3	3	1	1	2	1	3	3	0	N/A	0	2	N/A	2	0	21
Bammeke and Ndikom (2014)	2	3	3	3	1	2	2	3	0	0	N/A	0	2	N/A	0	0	21
Hyacinth et al. (2012)	0	3	2	0	2	2	2	0	0	1	N/A	1	2	N/A	0	3	18
Leo et al. (2020)	0	3	3	2	2	3	1	2	0	1	N/A	1	2	N/A	0	2	22
Okunowo and Smith-Okonu (2020)	0	3	3	3	2	2	2	2	0	0	N/A	0	0	N/A	0	0	17
Ndikom et al. (2020)	0	3	3	2	1	2	1	2	3	0	N/A	0	2	N/A	0	0	19

Criteria (scores 0 = not at all, 1 = very slightly, 2 = moderately, 3 = complete)

1 = explicit theoretical framework; 2 = statement of aims/objectives in main body of report; 3 = clear description of research setting; 4 = evidence of sample size considered in terms of analysis; 5 = representative sample of target group of a reasonable size; 6 = description of procedure for data collection; 7 = rationale for choice of data collection tool(s); 8 = detailed recruitment data; 9 = statistical assessment of reliability and validity of measurement tool(s) (quantitative only); 10 = fit between stated research question and method of data collection (quantitative only); 11 = fit between stated research question and format and content of data collection tool (e.g., interview schedule) (qualitative); 12 = fit between research question and method of analysis; 13 = good justification for analytical method selected; 14 = assessment of reliability of analytical process (qualitative only); 15 = evidence of user involvement in design; 16 = strengths and limitations critically discussed, N/A = Not applicable

### Analysis of the included studies

Table 3 shows the barriers that emerged from the thematic analysis and the number of studies in which they were reported. The barriers reported are lack of knowledge of cervical cancer and screening, screening is unnecessary, fear of screening outcome and procedure, financial constraints, negative misconception about cervical cancer and the screening, discrimination and stigmatization, health service factors, modesty, personal attributes of women, and cultural factors.

**Table 3:** Barriers to cervical cancer screening

Sn	Themes	No of studies	The proportion of respondents reporting a barrier in each study (%)														

			a	b	c	d	e	f	g	h	i	j	k	l	m	n	o
1.	**No knowledge of cervical** **cancer and screening**	13	NS	50.0	95.5	--	51.9	49.8	20.1	46.1	77.4	74.0	64.2	51.7	NS	57.1	--
2.	**Health service factors**																
	Difficulty in assessing screening	15	NS	42.1	0.8	65.0	21.1	3.9	35.0	11.6	35.5	70.0	27.7	35.1	--	14.3	--
	Lack of health education		NS	--	--	65.8	--	--	--	--	77.4	39.0	--	39.5	--	--	--
	No screening facility		--	59.9	--	--	--	--	--	--	--	70.0	--	37.1	--	--	NS
	Bad attitude of health workers		--	51.3	--	--	--	--	--	--	51.6	32.0	--	22.9	NS	--	NS
	Time constraint		--	48.0	--	--	13.2	--	--	--	62.9	26.0	--	--	--	--	NS
	Poor quality of health services		--	--	0.5	--	--	--	--	--	--	--	--	--	--	--	NS
	Not recommended by physician		--	--	--	69.2	--	--	--	5.4	--	47.0	41.4	--	--	--	--
3.	**Screening is unnecessary**																
	Lack of interest	10	NS	--	1.8	--	--	--	--	--	--	--	--	--	--	--	--
	Insignificant because of no cure		--	25.8	--	--	36.8	3.4	--	12.5	--	--	--	--	--	9.1	NS
	Absence of symptoms		--	--	--	--	51.6	32.0	51.2	--	--	--	--	--	NS	--	--
4.	**Fear**																
	Fear of positive result	12	NS	65.1	--	--	37.6	2.4	--	11.6	61.3	--	10.5	37.1	NS	--	--
	Fear of painful procedure		--	46.7	--	39.7	--	--	--	--	33.9	28.0	5.4	36.6	NS	--	NS
5.	**Financial constraint**	12	NS	45.4	0.8	64.5	19.6	5.5	45.2	5.4	35.5	39.0	12.3	31.7	--	--	--
6.	**Misconceptions**																
	Exposure to diseases	7	--	27.0	--	--	20.6	--	--	--	--	--	--	--	NS	--	--
	Cannot have the cancer		--	56.1	--	--	--	--	--	--	--	46.0	20.1	--	--	--	--
	For infected women only		--	--	--	--	--	--	--	--	22.6	--	--	--	--	--	--
	Avoid detection of diseases		--	--	--	--	--	--	35.0	--	--	--	--	--	--	--	--
7.	**Discrimination and stigma**	3	--	--	--	--	--	--	80.4	--	--	--	--	--	NS	--	NS
8.	**Modesty concerns**																
	Embarrassment	8	--	52.9	--	--	--	--	94.0	--	35.5	--	7.6	--	NS	1.9	--
	Violation of privacy		--	28.3	--	--	51.5	--	--	--	46.8	--	--	18.5	NS	--	--
9.	**Attributes of women**																
	Lack of education	9	NS	--	--	--	--	--	--	--	--	--	--	--	--	--	NS
	Need for spousal approval		--	51.0	--	--	--	--	--	--	56.5	--	4.2	22.4	NS	--	NS
	Religious belief		--	--	--	--	8.8	--	10.1	--	--	--	--	14.1	NS	--	NS
	Location of residence		--	--	--	--	--	--	--	--	--	--	--	--	NS	--	--
10.	**Cultural belief**	4	--	15.2	--	--	--	--	--	--	--	--	--	14.1	NS	--	NS

a = Ndikom and Ofi (2012), b = Titiloye et al. (2017), c = Abiodun et al. (2013), d = Amu et al (2017), e = Chigbu and Aniebue (2011), f = Nwankwo et al. (2011), g = Ogwunga et al. (2020), h = Ezem (2007), i = Amos and Awolude (2019), j = Bammeke and Ndikom (2014), k = Okunowo and Smith-Okonu (2020), l = Ndikom et al. (2020), m = Isa Modibbo et al. (2016), n = Hyacinth et al. (2012), o = Leo et al. (2020), NS = Not specified, -- = Not reported.

### Lack of knowledge of cervical cancer and screening

Lack of knowledge of cervical cancer and screening was reported as a barrier in 13 studies (Table 3). Three studies reported that most of their respondents were unaware and had no knowledge of cervical cancer and screening^19^,^21^,^25^. Although 10 studies reported a high level of awareness among their respondents, lack of adequate information about cervical cancer and screening methods was a significant barrier to the uptake of screening services amongtheir respondents^20^,^22^,^24^,^26^–^30^,^32^,^33^. Titiloye et al.^20^ and Chigbu and Aniebue^24^ reported that 73.8% and 55.2% of their respondents were aware of cervical cancer and screening. However, only 35.6% (20) and 49.0% (24) had satisfactory knowledge. Similarly, Isa Modibbo et al.^22^ reported that although most of their respondents had heard about cervical cancer, only one respondent attributed its cause to HPV.

### Health service factors

Numerous health service factors were reported as barriers in 15 studies (Table 3). Among these, difficulty in assessing screening^19^–^21^,^23^–^30^,^32^,^33^, poor orientation and screening recommendations^19^,^21^,^23^,^28^,^29^,^31^,^33^, no screening facility^20^,^29^,^31^,^33^, unfriendly attitude of healthcare providers^20^,^22^,^28^,^29^,^31^,^33^, poor quality of health services^21^,^31^, and time constraint^20^,^24^,^28^,^29^,^31^ were recorded.

### Screening is unnecessary

Cervical cancer screening was perceived to be insignificant by most of the respondents in 10 studies due to the absence of symptoms, lack of interest and belief that cervical cancer has no cure (Table 3). People usually do not bother about preventive services when they are healthy; thus, cervical screening is generally perceived as unnecessary^19^–^22^,^24^–^27^,^30^,^31^.

### Fear of screening outcome and procedure

Fatalistic view of cervical cancer diagnosis and fear of the screening procedure was stated as a barrier in 12 studies (Table 3). Some participants in a qualitative study stated that a positive result was presumed as a death warrant; thus, it is better not to undergo screening^19^. Similarly, the respondents in eight other studies reported that the fear of having a positive screening result hindered them from being screened^20^,^22^,^24^,^25^,^27^,^28^,^32^,^33^, while the fear of pain and discomfort during the screening procedure was a barrier in 8 studies^20^,^22^,^23^,^28^,^29^,^31^–^33^.

### Financial constraints

Financial constraint was also reported as a barrier to screening utilization (Table 3). The available screening services are not free^19^, and women usually prioritize their financial and social responsibilities due to economic constraints^34^,^35^. It was reported that screening services are unaffordable and expensive in 12 studies^19^–^21^,^23^–^29^,^32^,^33^.

### Misconception about cervical cancer and cervical cancer screening

Misconceptions and myths about cervical cancer can lead to conflicting perceptions about cervical cancer screening, as reported in Table 3. Some women reported that screening is meant for only promiscuous women^20^ and women with sexually transmitted infections (STIs)^28^ by 56.1% and 22.6%, while 46.0% and 20.1% in two other studies^29^,^32^ believed they cannot have cervical cancer. It was also reported that screening would expose women to STIs and other nosocomial infections in three studies^20^,^22^,^24^. However, the participants in Ogwunga et al. (2020) stated that they did not utilize screening to avoid detection of other diseases.

### Discrimination and Stigmatization

A feeling of stigmatization and discrimination poses a significant barrier to cervical screening uptake^22^,^26^,^31^. These deter women from accessing screening services because they fear discouraging comments from others. Stigma and discrimination associated with cervical cancer are linked to the sexually transmitted nature of the causative agent (HPV) and perceived immoral behaviour. Thus, some of the respondents expressed concerns about the confidentiality of their results^22^ (Table 3).

### Modesty

Embarrassment and concern for modesty were observed as a barrier in eight studies (Table 3). Some participants in five studies reported that cervical cancer screening would violate their privacy^20^,^22^,^24^,^28^,^33^,^20^,^24^. The respondents in three of the studies reported that the lack of female health workers in the screening facility hindered them from utilizing the service^22^,^28^,^33^. Besides, 5 studies reported that their respondents felt embarrassed to have any genital examination^20^,^22^,^26^,^28^,^30^,^32^.

### Personal attributes of women

Personal attributes refer to women's socio-demographic characteristics such as age, marital status, educational status, income level, and many more. Educational status was reported as a barrier in two studies^19^,^31^ (Table 3). In Ndikom and Ofi^33^ qualitative study, it was stated that only literates utilize screening services because those with low educational qualifications believed that “what you do not know cannot kill you.” The need for spousal approval was observed as a barrier in 6 studies^20^,^22^,^28^,^31^–^33^. Two of the studies revealed that they required their husbands' permission before being screened^20^,^22^ while 4 reported that they were discouraged by their husbands^28^,^31^–^33^. The location of residence was also reported as a barrier in a qualitative study; the respondents stated that awareness was limited to women residing in the city^22^. Religious belief was a barrier in five studies^22^,^24^,^26^,^31^,^33^ (Table 3). Some of the participants in three of the studies reported that they did not utilize cervical cancer screening because of their faith^24^ while the respondents in two other studies reported that their religion does not permit screening processes that involve exposure of the body^22^,^33^.

### Cultural factors

The culture of respondents was reported as a barrier in four studies (Table 3). One of the studies revealed that all the FGD participants in their study identified cultural modesty norms as a barrier to seeking cervical cancer screening^22^. However, only a small proportion of the respondents in two other studies reported that their culture forbids them from utilizing screening services^20^,^33^.

## Discussion

### Key findings

Several factors have been identified as barriers to cervical cancer screening utilization by Nigerian women. In this study, lack of adequate knowledge was observed as the primary barrier across the included studies; this is significantly associated with other factors highlighted in the studies, such as lack of interest and perceived low risk of susceptibility, fatalistic view, and misconceptions about cervical cancer screening. It is worthy of note that 61.3%^28^, 51.6%^24^, 46%^29^, 51.2%^26^, and 56.1%^20^ of the participants in five of the studies that reported a high level of awareness and knowledge of cervical cancer did not utilize screening due to fatalistic view of the disease^28^, absence of symptoms^24^,^26^, and perceived low risk of susceptibility^20^,^29^. Furthermore, 46.7% of the respondents in one of the studies stated that cervical screening would expose them to sexually transmitted diseases (STDs)^20^. These barriers were also observed in the studies that reported a low level of awareness among their respondents^19^,^21^,^23^,^25^. The negative effect of inadequate knowledge on screening utilization was observed in other studies. A study conducted in Uganda reported that women were generally uninformed about the benefits of screening, as such, fear of the screening procedure which is associated with perceived pain, misconceptions and fatalism was a major barrier to screening^36^. Likewise, negligence, absence of symptoms and fear of results were also reported in other studies conducted in sub-Sahara Africa^37^ and Latin America^38^. In addition, the feeling of being stigmatized or discriminated if diagnosed of cervical cancer was observed to be associated with inadequate knowledge of cervical cancer. This feeling is due to the sexually transmitted nature of the virus (HPV) that causes the disease^22^, as such, cervical cancer is linked to immoral behaviour. Negative perceptions about cervical screening by sexual partners, family members, and close friends can lead to avoidance of planning to utilize the screening services. Also, due to the negative perceptions about cervical cancer, disclosing a positive HPV or screening result to partners, relatives and friends might be frightening. This perception could interfere with the necessary treatment and supportive services needed by women with an abnormal result; hence, screening services are poorly utilized. Stigma and discrimination were also reported as a barrier to screening in other studies conducted in sub-Sahara Africa^36^,^37^. This result suggests that mere awareness of cervical cancer does not necessarily translate to the knowledge that can enhance preventive practices. Adequate information about cervical cancer through health education and medical sensitization can help promote preventive practices in Nigeria.

Numerous health service factors were also observed as a barrier to screening uptake, and this encompasses both the structure of the health facilities and the personnel. Lack of health education and insufficient medical advice is one of the significant barriers related to health service. Ndikom and Ofi (2012) in their qualitative study stated that cervical cancer orientation is low compared to the Human immunodeficiency virus (HIV). Similarly, most of the respondents in another study reported insufficient medical advice (69.2%) and lack of education as a barrier (65.8%) 23. Despite the severity and preventive nature of the disease, it is yet to be included as part of women's routine health education topic during gynaecological visits; also, cervical screening is not included as one of the routine tests for women in healthcare facilities. In Nigeria, a national cervical cancer screening program is absent^6^, coupled with a low referral of physicians^24^,^25^,^29^,^32^,^39^, thus screening is opportunistic^40^. The health service barriers reported in this study are consistent with the findings of a similar study conducted in countries with opportunistic screening programmes^38^. Studies have shown that the existence of a national screening program in developed countries has significantly reduced the burden of cervical cancer. In England, the incidence of cervical cancer decreased from 22 to 13 per 100,000 between 1972 to 2012 due to the introduction of the national cervical screening programme in 1988^41^ while in Australia, it was reported that the incidence and mortality was 10 and 2 per 100,000 in 2015^42^. Likewise, it has been reported that cervical cancer orientation and screening referral during gynaecological visits significantly improve screening uptake^25^,^31^,^43^.

Wastage of time is another factor associated with health service delivery in Nigeria. This constraint could be associated with the limited number of health workers and the structure of healthcare facilities. In most facilities, healthcare providers that render screening services might also be required to provide antenatal and family planning services, thus, overloaded with work and unable to attend to clients timely. Furthermore, screening services are not offered daily in most healthcare facilities; thus, women are only expected to visit the facilities when this service is available^38^. This scenario usually makes the screening process time-consuming and inconvenient for some women^20^,^28^,^29^,^31^. Proximity to screening facilities is also a significant barrier, especially for women living in communities with limited access to health care. Long distances to health facilities deter them from utilizing screening services because most of these services are offered at the tertiary healthcare level and private facilities that are only available in urban areas and usually require a high transportation cost 38. Hyacinth et al. (2012) reported that all the respondents screened in their study were at a secondary or tertiary health facility, while Adepoju et al. (2016) noted that the majority (87.1%) of the participants who utilized a free screening program were urban based. Poor accessibility has also been reported as a barrier in other studies 36–38. In addition to the lack of accessibility to screening facilities, some respondents in four studies 20,29,31,33 reported the non-existence of screening facilities as a barrier. Agurto et al. (2004) stated that this is not a factor in most studies conducted in developed countries. They noted that while this barrier seemed a single distinctive factor for women in low socioeconomic status in Latin America, the Hispanic women with low socioeconomic status in Canada do not report this because most of them have a physician whom they consult regularly, and the country has a national public health care system^38^. Worthy of note, while the lack of time due to competing demands, difficulty finding childcare, remembering to make and/or attend an appointment, transportation cost, and language barrier were the confounding factors to screening service accessibility among underserved groups (rural residents, immigrants, racial/ethnic minority groups, unemployed women, those who speak a language other than English) in the United States^44^; in Nigeria, the non-existence of screening facilities in most regions of the country poses a major challenge to screening accessibility.

Financial constraint is another factor that poses a barrier to the uptake of screening, and it was associated with the need for spousal approval in one study 22. The cost of screening is usually expensive, and most women cannot afford to participate even at a subsidized cost 39. There is a meagre budget for health support at all government levels, and less than 5% of the working population in the formal federal sector are enrolled in the NHIS 45, so out-of-pocket expenditure is a major financial source. The screening method available in the few facilities offering screening services in Nigeria is the Papanicolaou test and the average cost is estimated at US$16-$30 46,47 which is equivalent to N6,080-N11,400 based on the current exchange rate^48^. On the other hand, it is estimated that the average monthly cost of living for an individual in Nigeria amounted to N43,200 (US$113) and N137,600 (US$362) for a family while the minimum wage was increased to N30,000 (US $77) in 2019 from N18,000 (US$47)^49^. Furthermore, among the few with health insurance coverage, screening for cervical cancer is not covered by the NHIS^50^. Thus, with the high poverty level in Nigeria, women are forced to prioritize other expenses, thereby neglecting their health issues. It is therefore pertinent for the government to invest on saving the lives of women by establishing a free or subsidized national VIA and cryotherapy screening program in the country. In comparison with the cost of obtaining a Pap test, a recent economic analysis estimated that US$3.33-$37.58 (N1,265-N14,280) is required per woman to conduct a VIA and cryotherapy two-visit approach (cryotherapy would occur only at the district level) or US$7.31-$70.91 (N2,778-N26,946) for a single-visit approach (cryotherapy would be available at all facilities offering screening)^51^. Overall, approximately US$59 million would be required to purchase treatment equipment if cryotherapy were placed at every screening facility and about 20 million women would be screened over 10 years^51^. Likewise, a study conducted in a neighbouring West African country estimated a national annual program cost of US$0.6–4 million for VIA and treatment respectively while on the individual level, they estimated a cost of US$4.93-$14.75 (N1,873-N5,605) and US$47.26-$84.48 (N17,959-N32,102) per woman for VIA and treatment respectively^52^. This finding in Ghana suggests that a similar national program is feasible and could be more cost-effective considering the huge population of women in Nigeria who could benefit from the preventive program. However, studies have shown that providing highly subsidized or free screening services alone is not enough to improve its uptake; this must be done simultaneously with effective awareness creation and improved service accessibility^38^–^40^,^53^.

The sense of modesty and embarrassment also deter women from accessing screening services because it involves examining the reproductive organ, and women generally perceive this as “violating their privacy”^20^,^24^. Culturally, the cervix is perceived as a private part of the body in Africa^54^, which is not an exception in Nigeria. Women tend to shy away from gynaecological examinations, mostly when provided by a male healthcare worker^22^,^28^,^33^. Embarrassment has also been reported as a factor inhibiting screening uptake in other studies and was associated with lack of privacy in the screening facility and modesty concerns^38^,^55^. In this study, it was observed that religion could also be associated with this factor; all the Muslim participants in a qualitative study emphasized that it is against their religion to expose their bodies. However, they stated that they were few exceptions that involve illnesses and preventive measures. Also, most of the study participants preferred the samples to be taken at a health facility by a healthcare provider instead of self-sampling at home because they believed inadequate samples of low quality might be collected^22^. Although the above suggests that improved knowledge of cervical cancer screening benefits will reduce the effect of modesty and embarrassment on screening uptake, it further highlights the need to educate women on the use of self-sampling methods. Studies have shown that the use of self-sampling which involves women swabbing their own cervical tissue for HPV testing can improve attendance to cervical cancer screening programme with overall acceptability greater than 80% following an audio-visual presentation and in-person instructions^56^,^57^. In furtherance, the impact of improved knowledge of screening benefits on screening uptake was observed in a study where 71% of the unscreened participants in the intervention group participated in screening after a 6 months intervention while only 22% of the control group utilized screening without an intervention^58^. In addition, screening facilities should be designed to ensure adequate privacy during the procedure and screening campaigns should be conducted in appropriate locations such as primary health centres or decentralized mobile clinics rather than schools as reported in Agurto et al. (2004) study.

Some personal attributes of women, such as educational level, location of residence, income level and religion were reported as barriers to screening. The respondents in two studies stated that illiteracy was a barrier to screening utilization though no association was established^19^,^31^. Similarly, it was reported in 4 studies that educational level has a significant relationship with screening utilization^24^,^25^,^29^,^30^ though the studies did not control for other parameters, while one study contradicted the relationship between education and screening uptake^31^, but no cause was established. The economic status of women was also reported as a screening barrier. Three studies reported that a high-income level significantly increased screening uptake^28^,^29^,^33^. The location of residence was also reported to be associated with the utilization of cervical screening. In Nwankwo et al.^25^, it was reported that 82.4% of the respondents that had been screened resided in the urban areas while 17.6% resided in the rural areas; this was also the case in Adepoju et al.^53^. The location of residence was also reported as a factor influencing the increase in cervical cancer and screening awareness in a qualitative study by Isa Modibbo et al.^22^. Likewise, Chigbu and Aniebue (2011) reported that 63.5% of those with knowledge of cervical cancer screening resided in the urban while 36.5% resided in the rural areas. In addition, Isa Modibbo et al.^22^ reported that there was a low level of awareness of cervical cancer and a higher prevalence of reluctance to engage with the healthcare system among the Muslim participants. This reluctance could be attributed to the low availability of public health campaigns in religious gatherings, especially among Muslims. While the reports of these studies are consistent with previous research that has shown that women from low socioeconomic background and rural areas are less likely to utilize screening^59^,^60^; the association between cervical screening utilization and the socioeconomic characteristics of Nigerian women in addition to their religious beliefs were not well established in the studies. This finding suggests the need for further studies on the sociodemographic determinants of cervical screening among women in Nigeria. In furtherance, the use of Lay health advisors (trusted individuals from the same communities who have been trained to provide education, emotional and logistical support and advice), and in reach (patients served by a local healthcare centre are found through the facility lists and visited at home for sensitization) educational programmes can help reduce the knowledge gap that exists among women in Nigeria^61^.

## Strength and limitation

This study offers a comprehensive synthesis of the barriers to cervical cancer screening among women in Nigeria and the first to provide an overview of the issues across the country.

Our major limitation was the unstandardized quality of the studies included and the limited number of studies that assessed the barriers to cervical cancer screening among women as the primary outcome. Secondly, the included studies were conducted in three geopolitical regions; eight studies were in the Southwestern geographical zones, 4 in the Southeast, 2 in the North-central, while one was conducted in both the Southwest and North-central region (Figure 2), thus, the information provided may not be generalized to other zones.

## The implication for future research

The limited generalisability of the findings of this study to other zones not included in the study indicates paucity of literature on this research topic in some parts of the country (Fig. 2). Understanding the barriers to cervical cancer screening across the various states and zones in the country is required to improve screening uptake, thereby reducing the disease burden in Nigeria. Therefore, there is a need for broader research coverage on the barriers to screening uptake in other parts of the country, especially the South-south, Northeast, and Northwest geopolitical zones. Secondly, future research should provide more evidence on the association between women's sociodemographic characteristics and cervical cancer screening awareness and utilization in Nigeria.

## Conclusion

This study offers a synthesis of the factors inhibiting cervical screening uptake among women in Nigeria. Amongst the barriers enumerated, lack of adequate cervical cancer and screening information was observed as the major hindrance to screening; this factor is strongly associated with the numerous misconceptions and negative perceptions about the disease. Hence, there is a crucial need to improve medical sensitization on cervical cancer and the benefits of screening in Nigeria. The study also highlights the need for further assessment of the sociodemographic determinants of cervical cancer screening uptake in Nigeria.

The National health policymakers should formulate policies to ensure that;

- The Ministry of Health incorporate cervical cancer education as one of the health topics provided by health workers during women's gynaecological visits.
- The Ministry of Education in collaboration with the Ministry of Health should implement periodic cervical cancer sensitization for students in all secondary and tertiary institutions.
- All Local Government health councils should coordinate and involve community and religious leaders in periodic cervical cancer peer education programs for their populace.
- The Federal Government should implement a free or subsidized national VIA and cryotherapy screening program in primary healthcare facilities, decentralized mobile health clinics, secondary, and tertiary level health institutions through the Local Government health councils and the Ministry of Health.
- The Ministry of Health should incorporate cervical screening as a routine test for women in any healthcare setting, and healthcare workers should make screening recommendations to women.
- Healthcare providers should be trained regularly on screening procedures and patient-centred care.
